# Rea regulates microglial polarization and attenuates neuronal apoptosis via inhibition of the NF‐κB and MAPK signalings for spinal cord injury repair

**DOI:** 10.1111/jcmm.16220

**Published:** 2020-12-25

**Authors:** Shining Xiao, Chenggui Wang, Quanming Yang, Haibin Xu, Jinwei Lu, Kan Xu

**Affiliations:** ^1^ Department of Orthopedic Surgery School of Medicine The Second Affiliated Hospital Zhejiang University Hangzhou China

**Keywords:** microglia polarization, neuronal apoptosis, Rehmannioside A, spinal cord injury

## Abstract

Inflammation and neuronal apoptosis aggravate the secondary damage after spinal cord injury (SCI). Rehmannioside A (Rea) is a bioactive herbal extract isolated from *Rehmanniae radix* with low toxicity and neuroprotection effects. Rea treatment inhibited the release of pro‐inflammatory mediators from microglial cells, and promoted M2 polarization in vitro, which in turn protected the co‐cultured neurons from apoptosis via suppression of the NF‐κB and MAPK signalling pathways. Furthermore, daily intraperitoneal injections of 80 mg/kg Rea into a rat model of SCI significantly improved the behavioural and histological indices, promoted M2 microglial polarization, alleviated neuronal apoptosis, and increased motor function recovery. Therefore, Rea is a promising therapeutic option for SCI and should be clinically explored.

## 1 INTRODUCTION

Spinal cord injury (SCI) is a refractory, debilitating disease which can lead to the loss of sensory and motor functions in the affected parts.^1^, ^2^ Apart from the mechanical and structural damage caused by physical trauma,^3^, ^4^ SCI is also accompanied by pathological changes like inflammation, apoptosis, ischaemia and local oedema, which are collectively known as secondary injury.^5^, ^6^, ^7^, ^8^ Microglial polarization is a key mechanism of the secondary inflammatory injury following SCI and an effective therapeutic target since regulation of microglial polarization can mitigate inflammation and reversing secondary inflammatory injury.^9^, ^10^ Neuroinflammation is driven by the activated M1 microglia, leading to neuronal apoptosis and damage to the central nervous system.^11^, ^12^ The severity of inflammation after SCI significantly affects motor function recovery^13^, ^14^ via loss of neurons.^15^ Therefore, we recommend that the treatment strategy of SCI should emphasize on inhibiting the inflammatory response and improving the neuronal microenvironment in order to reduce apoptosis rates.

The inflammatory response is primarily mediated by the NF‐κB^16^ and MAPK^17^, ^18^ signalling pathways. NF‐κB transcriptionally activates pro‐inflammatory factors including cyclooxygenase (COX)‐2, inducible nitric oxide synthase (iNOS), interleukin‐6 (IL‐6), tumour necrosis factor‐α (TNF‐α), IL‐1β etc,^19^, ^20^ and is a key factor in chronic diseases with an inflammatory basis.^21^, ^22^ In addition, the MAPK signalling pathway modulates the expression levels of genes related to inflammation, apoptosis and cellular differentiation^23^ by activating the c‐Jun N‐terminal kinases (JNKs), p38 and extracellular signal‐related kinases (ERK) cascades.^24^ The activated transcription factors then influence the level of relevant genes which affect the expression of pro‐inflammatory factors and thus lead to neuroinflammation. Therefore, a viable therapeutic strategy against SCI is to inhibit neuronal inflammation and apoptosis by inactivating NF‐κB and MAPK signalling.

**FIGURE 1:**
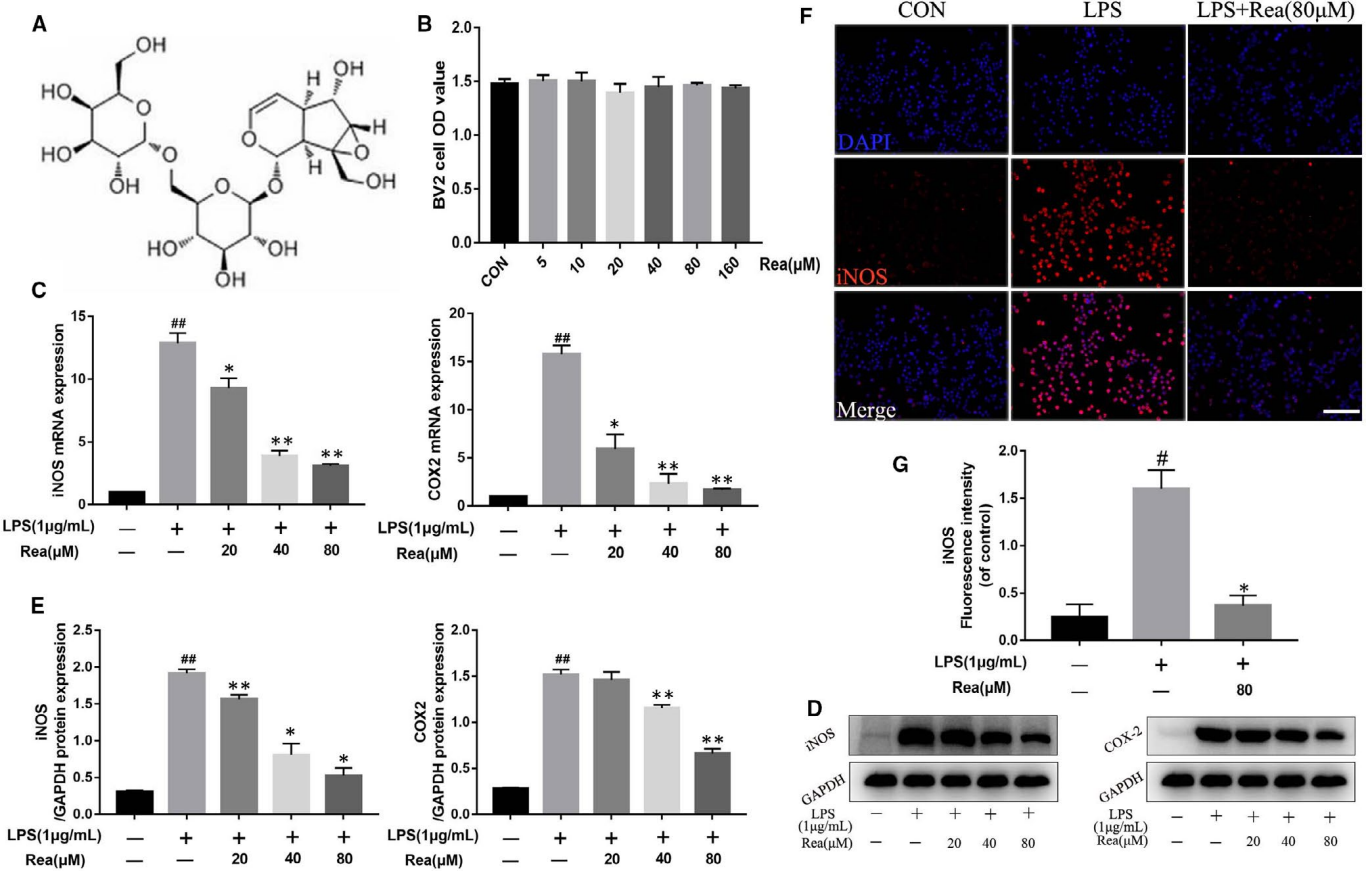
Rea inhibited the release of pro‐inflammatory mediators in BV2 cells. (A) The chemical structure of Rehmannioside A. (B) The cell viability of BV2 cells treated with Rea at different concentrations for 24 h and was evaluated by CCK‐8 assay. (C) The BV2 cells were overnight pre‐treated with or without Rea and stimulated with LPS for another 24 h. The result of qRT‐PCR for iNOS and COX‐2 gene expression levels in BV2 cells. (D, E) Western blot results and quantification dataof iNOS and COX‐2 in BV2 cells treated above. (F, G) Immunofluorescence staining and quantification analysis for iNOS in BV2 cells treated above. Scale bar = 100 μm. #*P* < .05, ##*P* < .01 vs the BV2 cells were untreated. *P < .05, **P < .01 vs the BV2 cells were treated with LPS alone

Rehmannioside A (Rea, C_21_H_32_O_15_, Figure 1A) is a neuroprotective compound^25^, ^26^ isolated from *Rehmanniae radix*, an herb used in various Chinese medicine formulations. It remains to be elucidated whether Rea can promote functional recovery after SCI.

In the present study, we assessed the neuroprotective characteristics of Rea both in vitro and in vivo, along with pharmacological effects and underlying mechanisms. Our results indicated that Rea inhibited both NF‐κB and MAPK signalling pathways and mitigated inflammation by promoting M2 (anti‐inflammatory phenotype) polarization of the microglia, which eventually attenuated neuronal apoptosis. In the animal model of SCI as well, daily intraperitoneal injection of 80 mg/kg Rea significantly improved the behavioural and histological indices, and accelerated recovery of hindlimb motion. Overall, our experimental data indicated that Rea enhanced the polarization of M2 microglia and reduced neuronal apoptosis and promoted functional recovery through the regulation of MAPK and NF‐κB signals after SCI. Thus, Rea is a promising drug for SCI treatment and ought to be investigated further.

## 2 MATERIALS AND METHODS

### 2.1 Reagents and antibodies

Rehmannioside A (purity > 98% by HPLC) was obtained from HUIJIA BIOTECHNOLOGY (Xiamen, China) and dissolved in DMSO. Antibodies specific for IκBα, phosphorylated‐IκBα (p‐IκBα), NF‐κB p65 (p65), p‐NF‐κB p65(p‐p65), p38, p‐p38, ERK, p‐ERK, JNK, p‐JNK, iNOS, COX‐2, Arg‐1, Cleaved caspase 3 (C‐caspase3), Bcl‐2, BAX, MAP2 and GFAP were purchased from Cell Signaling Technology. Antibodies against CD68, NF‐200, NeuN and Lipopolysaccharide (LPS) were bought from Abcam, and the anti‐iNOS antibody was from Proteintech. Calcium fluorescein‐AM/PI was provided by Keygen Biotech.

### 2.2 Establishment of SCI model and treatment regimen

Thirty‐six female SD rats (200‐250 g, SLAC Laboratory Animal Company) were randomized into the Sham‐operated, SCI and SCI + Rea groups. To induce SCI, the animals were anaesthetized via intraperitoneal injection of 1% (w/v) pentobarbital sodium (40 mg/kg), and the muscles tissues around the T9 spinous process were separated. The spinal cord was then exposed by T9 laminectomy and clamped with vascular clamp (15 g force, Oscar) for 1 minute to induce SCI. The Sham‐operated rats underwent the same operation except for spinal cord compression. The bladder of the SCI model was manually emptied twice a day. In addition, 80 mg/kg Rea was injected intraperitoneally into the treatment group daily, and the other animals were injected with the same amount of normal saline. All protocols were consistent with the Animal Care and Use Committee of the Second Affiliated Hospital of Zhejiang University.

### 2.3 Functional behaviour evaluation

The locomotive function was assessed on days 0, 1, 3, 7, 14 and 28 post‐SCI with the Basso, Beattie and Bresnahan (BBB) scale and footprint analysis. The BBB rating scale ranged from 0 (complete paralysis of hind limbs) to 21 (normal movement).^27^ Each rat was observed individually for 5 minutes and the BBB score was recorded. Footprint analysis was performed by immersing the hindlimb in blue dye and the forelimb in red dye,^28^ and the rats were allowed to walk through a pipeline lined with a white paper (10 cm width and 100 cm length). The resulting footprints are evaluated. All tests were conducted by three independent investigators in a blinded manner.

### 2.4 Cell culture and co‐culture

BV2 and PC12 cells were cultured in high glucose DMEM supplemented with 10% foetal bovine serum (FBS), streptomycin (100 μg/mL) and penicillin (100 U/mL) at 37°C under 5% CO_2_. BV2 cells were pre‐treated overnight with Rea (0, 20, 40 and 80 μmol/L) and then incubated for 24 hours with or without 1 μg/mL LPS. For the co‐culture system, BV2 cells pre‐treated overnight with/out 80 μmol/L Rea, and then BV2 cells with/out LPS stimulation were rinsed with PBS to eliminate the effects of remaining LPS and Rea. Next, BV2 cells were seeded into inserts (pore size = 0.4 μm; Corning). The latter were placed on the PC12 monolayer at the bottom of the well and cultured for 24 hours.

### 2.5 Cell viability assay

Cell viability was evaluated using the CCK‐8 (Beyotime) kit according to the manufacturer's instructions. Briefly, BV2 cells were seeded in 96‐well plates at the density of 10 000/well, and with 0, 5, 10, 20, 40, 80 or 160 μmol/L Rea for 24 hours. In addition, BV2 cells pre‐treated overnight with Rea (0 or 80 μmol/L) and then with LPS (0 or 1 μg/mL) for 24 hours were co‐cultured with PC12 as described. Ten microlitres CCK‐8 reagent was added to each well, and the absorbance at 450 nm was measured after a 2 hours incubation.

### 2.6 Reverse transcription quantitative polymerase chain reaction (RT‐qPCR)

RNA was extracted from the suitably treated cells using Trizol reagent (Invitrogen) according to the manufacturer's instructions, and RT‐qPCR was performed as previously described.^29^ Each experiment was repeated thrice, and the relative gene expression levels were calculated using the 2^−ΔΔCt^ method. The primer sequences are shown in Table 1.

**TABLE 1:** Primers used for Quantitative Real‐Time PCR analysis

Gene	Forward	Reverse
iNOS	GTTCTCAGCCCAACAATACAAGA	GTGGACGGGTCGATGTCAC
COX‐2	TGAGCAACTATTCCAAACCAGC	GCACGTAGTCTTCGATCACTATC
TNF‐α	CAGGCGGTGCCTATGTCTC	CGATCACCCCGAAGTTCAGTAG
IL‐1β	GCAACTGTTCCTGAACTCAACT	ATCTTTTGGGGTCCGTCAACT
Arg‐1	GGGAAGGTAATCATAAGCCAGA	CCCAGATGACTTT TATGCGATG
IL‐10	TTACCTGGTAGAAGTGATGCCC	GACACCTTGGTCTTGGAGCTTA
β‐actin	GGCTGTATTCCCCTCCATCG	CCAGTTGGTAACAATGCCATGT

### 2.7 Calcein AM/propidium iodide (PI) staining

Apoptosis was evaluated by calcein AM/PI staining as per established protocols. Briefly, PC12 cells were seeded in a 6‐well plate, and co‐cultured as described with BV2 cells that were pre‐treated overnight with Rea (0 or 80 μmol/L), followed by LPS (0 or 1 μg/mL) for 24 hours. After co‐culturing for 24 hours, the medium was removed, and the PC12 cells were rinsed thrice with PBS. The cells were then incubated with calcein AM/PI at 37°C in the dark for 30 minutes. The stained cells were observed under a fluorescence microscope (Olympus).

### 2.8 Immunofluorescence Staining

In vitro study, BV2 cells was pre‐treated overnight with or without Rea (80 μmol/L) before stimulation with or without 1 μg/mL LPS for 24 hours. The cells were rinsed gently with PBS, fixed with 4% paraformaldehyde (PFA) at room temperature for 15 minutes, and permeabilized with 0.25% Triton X‐100 for 15 minutes. After blocking with 5% BSA for 60 minutes at 37°C, the cells were incubated overnight with the anti‐Arg‐1 (1:200) and anti‐iNOS (1:300) antibodies at 4°C. The cells were then rinsed with PBS and incubated with the specific secondary antibodies (Beyotime) at 37°C for 1 hour, followed by DAPI (Invitrogen) counterstaining for 5 minutes. The stained cells were observed under a fluorescence microscope (Olympus).

For vivo study, the differentially treated rats were perfused with 0.9% sodium chloride and 4% paraformaldehyde (PFA) after anaesthetization, and a segment of the spinal cord was cut approximately 1 cm on each side of the damaged region. Then, the tissues were fixed in 4% PFA for 48 hours, dehydrated and embedded in paraffin. Longitudinal 5μm‐thick sections were cut from the paraffin blocks, dewaxed and boiled in citrate buffer for antigen retrieval as previously described.^30^ After blocking with 5% BSA at 37°C for 1 hour, the tissue sections were incubated overnight with the primary antibodies against GFAP (1:300), CD68 (1:500), Arg‐1 (1:50), MAP2 (1:200) and NF‐200 (1:500) at 4°C. The following steps were same as above.

### 2.9 Western blotting

Total proteins were isolated from the cells and spinal cord tissues and quantified using the BCA reagent. Equal amount of protein per sample were loaded on 10% (for probing p65, p‐p65, IκBα, p‐IκBα, ERK, p‐ERK, p38, p‐p38, JNK, p‐JNK, iNOS, COX‐2, Arg‐1, NeuN, NF‐200 and MAP2) or 12% (for probing BAX, Cleaved caspase 3 and Bcl‐2) SDS‐polyacrylamide gels for electrophoresis. GAPDH was used as the internal control for all proteins. The bands were transferred to a PVDF membrane and incubated overnight with primary antibodies targeting iNOS, COX‐2, Arg‐1, Cleaved caspase 3, BAX, Bcl‐2, IκBα, p‐IκBα, p65, p‐p65, ERK, p‐ERK, p38, p‐p38, JNK, p‐JNK, NeuN, CD68, NF‐200, MAP2 and GAPDH proteins, followed by secondary antibody for 2 hours. The positive bands were detected using an image processing system (Bio‐Rad) and quantified by ImageJ software.

### 2.10 Histological staining

The tissue sections were processed as described and stained with haematoxylin and eosin (H&E) and observed under a light microscope (Olympus).

### 2.11 Statistical analysis

All data were presented as the mean ± standard deviation. The different groups were compared by one‐way analysis of variance and Tukey's post‐hoc test using GraphPad Prism 7.0. *P* < .05 was considered statistically significant.

## 3 RESULTS

### 3.1 Rea alleviates pro‐inflammatory mediators release from LPS‐induced BV2 cells

BV2 cells treated with different concentrations of Rea did not show any significant reduction in viability, indicating its low cytotoxicity at doses less than 160 μmol/L (Figure 1B). We next studied the effect of Rea on the secretion of pro‐inflammatory mediators from BV2 cells in vitro. BV2 cells were pre‐treated overnight with Rea (0, 20, 40, or 80 μmol/L) and then treated for 24 hours with or without LPS (1 μg/mL). As shown in the Figure 1C‐E, LPS stimulation significantly increased the levels of COX‐2 and iNOS mRNA and proteins compared to that in the untreated group (CON group). However, iNOS and COX‐2 production decreased significantly upon Rea treatment compared to the LPS group. In addition, expressions of iNOS and COX‐2 were significantly inhibited in a dose‐dependent manner, which were similar to those of the immunofluorescence expression of iNOS (Figure 1F,G). These data demonstrated that LPS up‐regulated pro‐inflammatory mediators in BV2 cells, which was attenuated by Rea pre‐treatment.

### 3.2 Rea promotes M2 polarization and suppresses neuronal apoptosis in vitro

**FIGURE 2:**
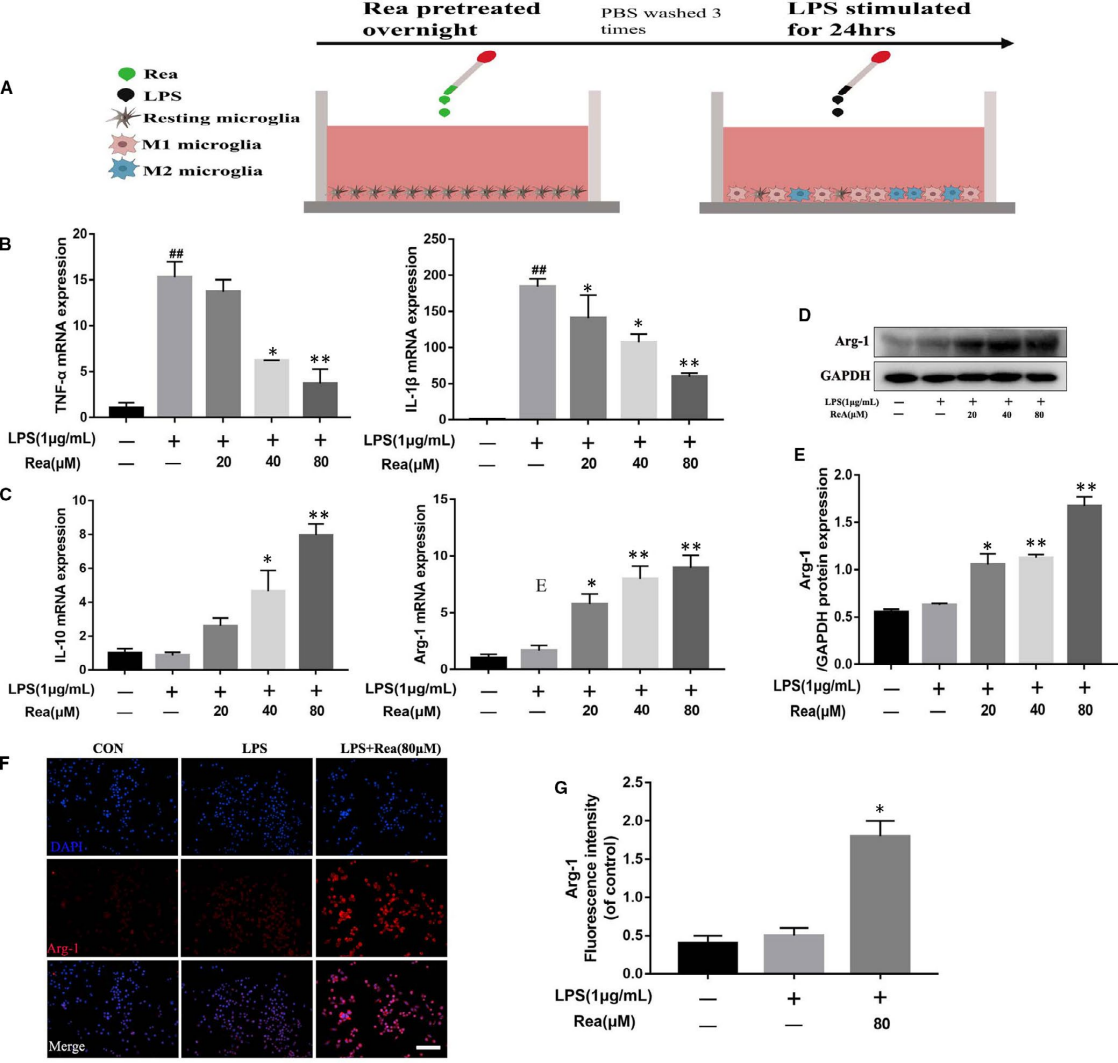
Rea promotes M2 polarization in microglia. (A) Schematic of BV2 cell treatments. BV2 cells were overnight pre‐treated with Rea, followed by washing three times and stimulation with LPS for 24 h. (B, C) The result of qRT‐PCR for TNF‐α, IL‐1β, Arg‐1 and IL‐4 gene expression levels in BV2 cells treated above. (D, E) Western blot and quantification analysis results of Arg‐1 in BV2 cells treated above. (F, G) Immunofluorescence staining and quantification analysis for Arg‐1 in BV2 cells. Scale bar = 100 μm. Data are presented as the mean ± SD from three independent experiments. #*P* < .05, ##*P* < .01 versus the BV2 cells were untreated. *P < .05, **P < .01 versus the BV2 cells were treated with LPS alone

We next assessed the effect of Rea on microglial polarization by evaluating the mRNA expression levels of M1 (pro‐inflammatory phenotype) and M2 (anti‐inflammatory phenotype) polarized microglial cells under Rea treatment (Figure 2A). Rea significantly inhibited the release of TNF‐α and IL‐1β in LPS‐induced microglial cells (Figure 2B), and enhanced the expression of M2 microglial markers like Arg‐1 and IL‐10 (Figure 2C). In addition, Arg‐1 protein levels also increased significantly after Rea treatment in a dose‐dependent manner (Figure 2D,E). Likewise, compared to the LPS‐induced group, the immunofluorescence intensity of Arg1 greater in the Rea‐treated BV2 cells (Figure 2F,G). These results indicate that Rea facilitates M2 polarization and decreases M1 polarization of microglia.

**FIGURE 3:**
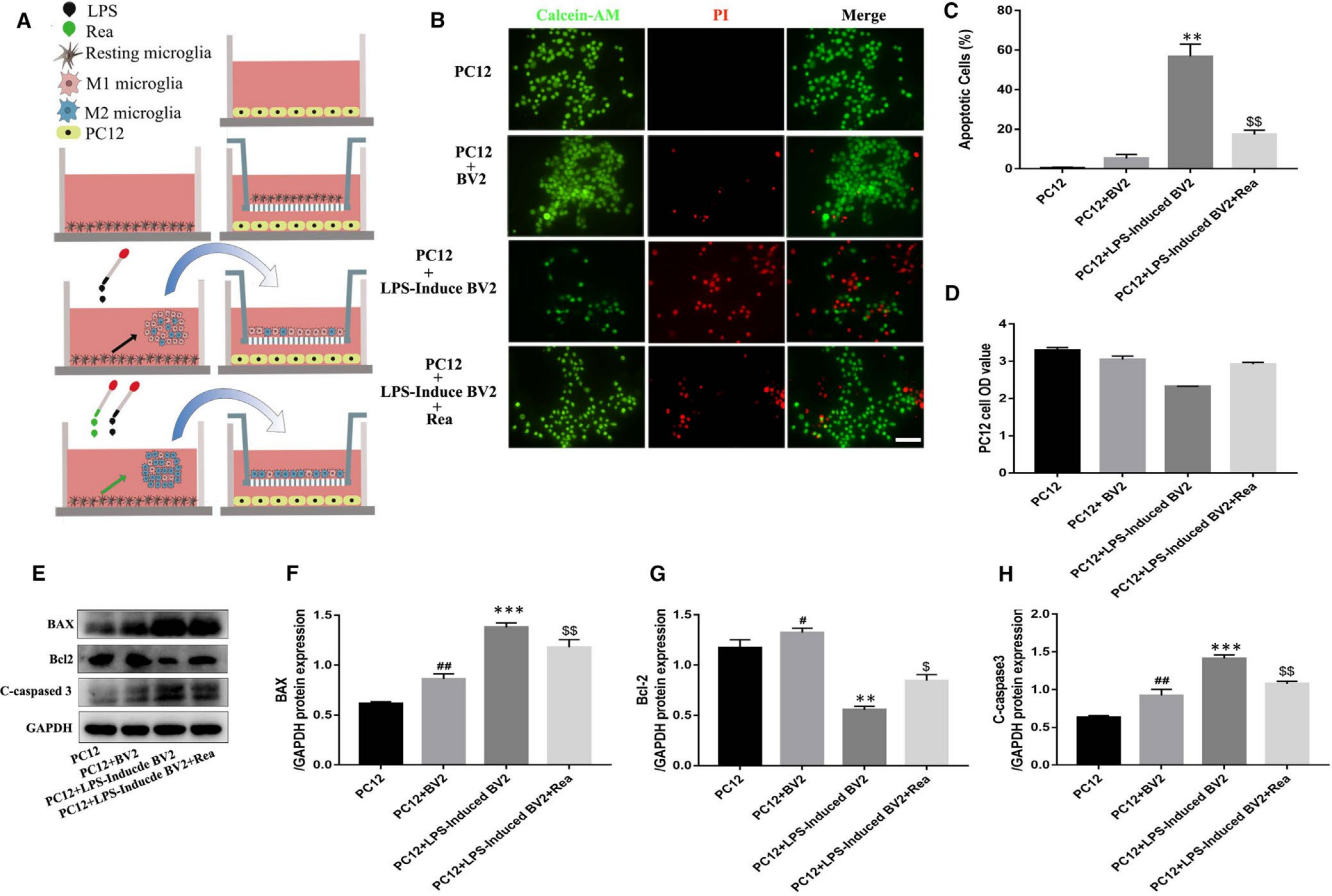
The anti‐apoptotic effect of Rea in co‐culture system of BV2 and PC12 cells. PC12 cells were untreated. BV2 cells were untreated, or treated with LPS (1 μg/mL), or pre‐treated overnight with Rea (80 μmol/L), and stimulation with LPS for 24 h, and then co‐cultured with PC12 cells. (A) Schematic showing how Rea attenuates neuron apoptosis and the relationship between BV2 cells and PC12 cells. (B, C) Calcein AM/PI double staining and quantification analysis for cell apoptosis in each group as treated above. Scale bar = 50 μm. (D) The cytotoxicity of BV2 cells on PC12 cells was detected by CCK8 assay in co‐culture system after 24 h. (E‐H) Western blot expression and quantification analysis of BAX, Bcl‐2 and cleaved caspase 3 were detected by in co‐culture system after PC12 cells treated above. Data are presented as the mean ± SD from three independent experiments. #*P* < .05, ##*P* < .01 vs the PC12 group. *P < .05, **P < .01, ***P < .001 vs the PC12 + BV2 group. $*P* < .05, $$*P* < .01 vs the PC12 + LPS‐Induced BV2 group

To determine the impact of Rea on neuronal apoptosis induced by the activated M1 microglia, we co‐cultured the differentially treated BV2 cells with the PC12 neuronal cell line (Figure 3A). The apoptosis rate in the monocultured PC12 cells was almost zero and increased marginally to 5.8% when cultured with the untreated BV2 cells. In contrast, the percentage of apoptotic PC12 cells markedly increased to 58.5% in the presence of the LPS‐stimulated microglia, whereas pre‐treatment of the BV2 cells with Rea resulted in a significant decrease to 18.3% (Figure 3B,C). Similar trends were seen with the viability of the co‐cultured neurons (Figure 3D). Furthermore, the pro‐apoptotic markers like cleaved caspase 3 and BAX were significantly up‐regulated in the PC12 cells co‐cultured with the LPS‐induced microglia, whereas the anti‐apoptotic protein Bcl‐2 was inhibited. The expressions of all proteins were restored in the presence of the Rea pre‐treated BV2 cells (Figure 3E‐H). Taken together, Rea prevents neuronal apoptosis by facilitating M2 polarization of microglia and mitigating inflammatory damage.

### 3.3 Rea suppresses NF‐κB and MAPK signalling pathways

**FIGURE 4:**
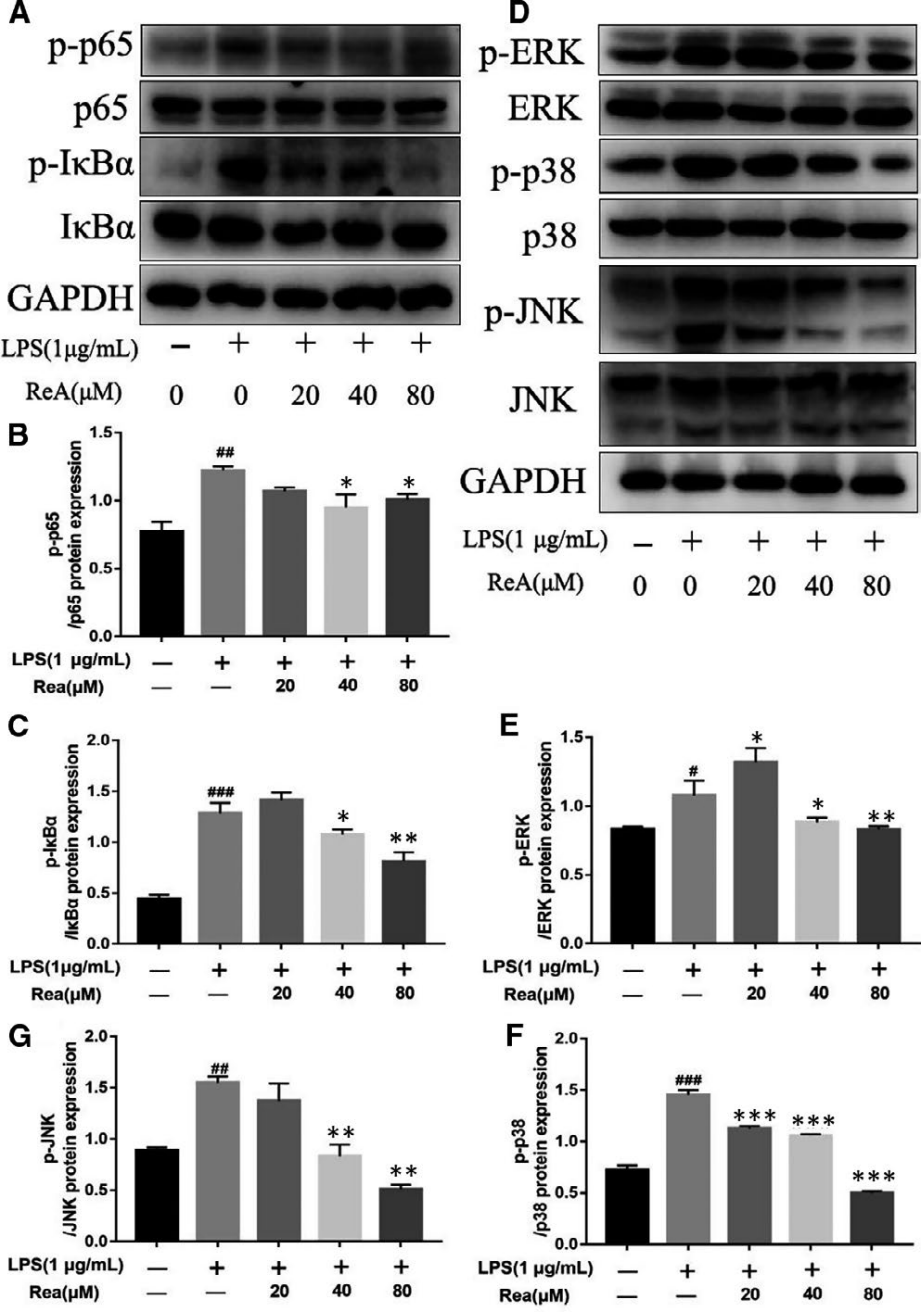
Rea treatment suppresses NF‐κB and MAPK signalling pathways in vitro. (A‐C) Western Blot protein expressions and quantification data of p65, p‐p65, IκBα and p‐IκBα was detected by Western blot in BV2 cells. (D‐G) Western Blot protein expressions and quantification data of ERK, p‐ERK, p38, p‐p38, JNK and p‐JNK was detected by Western blot in BV2 cells. Data are presented as the mean ± SD from three independent experiments. #*P* < .05, ##*P* < .01, ###*P* < .001 vs the BV2 cells were untreated *P < .05, **P < .01, ***P < .001, vs the BV2 cells were treated with LPS alone

Considering the importance of NF‐κB and MAPK signalling pathways in the inflammatory response, we next determined the effect of Rea on LPS‐induced NF‐κB activation and MAPK signal transduction. Compared to the untreated group, LPS activated the NF‐κB and MAPK signalling pathways in the microglia (Figure 4A,D), as indicated by the increased levels of phosphorylated p65, IκBα, JNK, ERK and p38. BV2 cells were then treated overnight with Rea at the concentration of 0, 20, 40 or 80 μmol/L and then stimulated with LPS for 24 hours. Rea treatment significantly down‐regulated these factors in a dose‐dependent manner. Thus, the neuroprotective effects of Rea can be attributed to the inactivation of the pro‐inflammatory and pro‐apoptotic NF‐κB and MAPK pathways.

### 3.4 Rea facilitates the recovery of motor function after SCI

**FIGURE 5:**
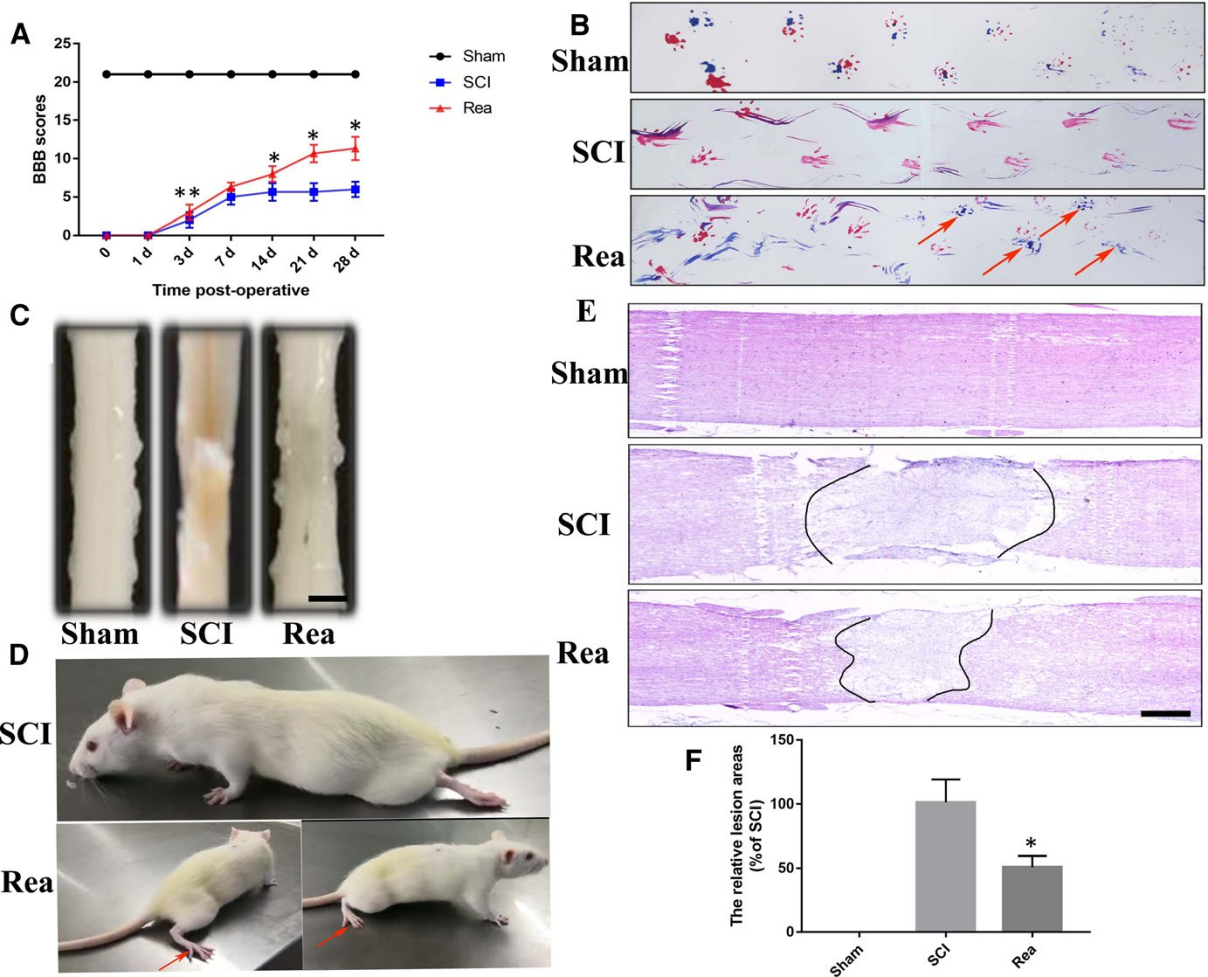
Rea improved pathology and motor function after SCI. (A) The Basso, Beattie, and Bresnahan (BBB) limb function scores at different times after spinal cord injury. (B) Representative footprints of an animal walking 28 days after SCI. Red: front paw print; blue: hind paw print. The red arrow shows the footprints of the fore‐and hind paws almost overlapping. (C) Gross morphology of spinal sections. Scale bar = 200μm. (D) Photos of activity of rats, red arrow showing hind limb landing on the ground. (E) Representative images from H&E staining at 28 days post‐injury. The dotted line indicates border of lesion site. Scale bar = 500 μm. (F) Quantification of the lesion cavity area of the spinal cord from H&E staining. Data are presented as the mean ± SD from three independent experiments. *P < .05 vs the SCI group

We performed behavioural and pathological measurements to assess functional recovery after SCI with or without Rea treatment. The behavioural analysis was based on the Basso Beattie Bresnahan (BBB) motor scores and footprint test. Following SCI induction, the rats showed lax palsy and recovered in a time‐dependent manner. However, the mean BBB score of the Rea‐treated rats was 11 compared to only 6 in the untreated SCI group (Figure 5A), indicating that Rea accelerates recovery after SCI. Consistent with this, the untreated rats showed obvious dragging of the hindlimbs in the footprint test (Figure 5B) whereas the Rea‐treated group partially recovered the coordination of front and hind limbs on day 28 after SCI (Figure 5B,D, red arrow). Furthermore, histological examination of the injured spinal cord showed numerous cavities in the longitudinal plane (Figure 5E), which were significantly narrowed upon Rea treatment, which is consistent with Figure 5C. In conclusion, Rea can accelerate the recovery of motor function in rats after SCI, likely by inhibiting the release of inflammatory factors from the activated microglia, and increasing neuronal survival and tissue regeneration.

### 3.5 Rea boosts M2 polarization and mitigates inflammation and neuronal apoptosis after SCI

**FIGURE 6:**
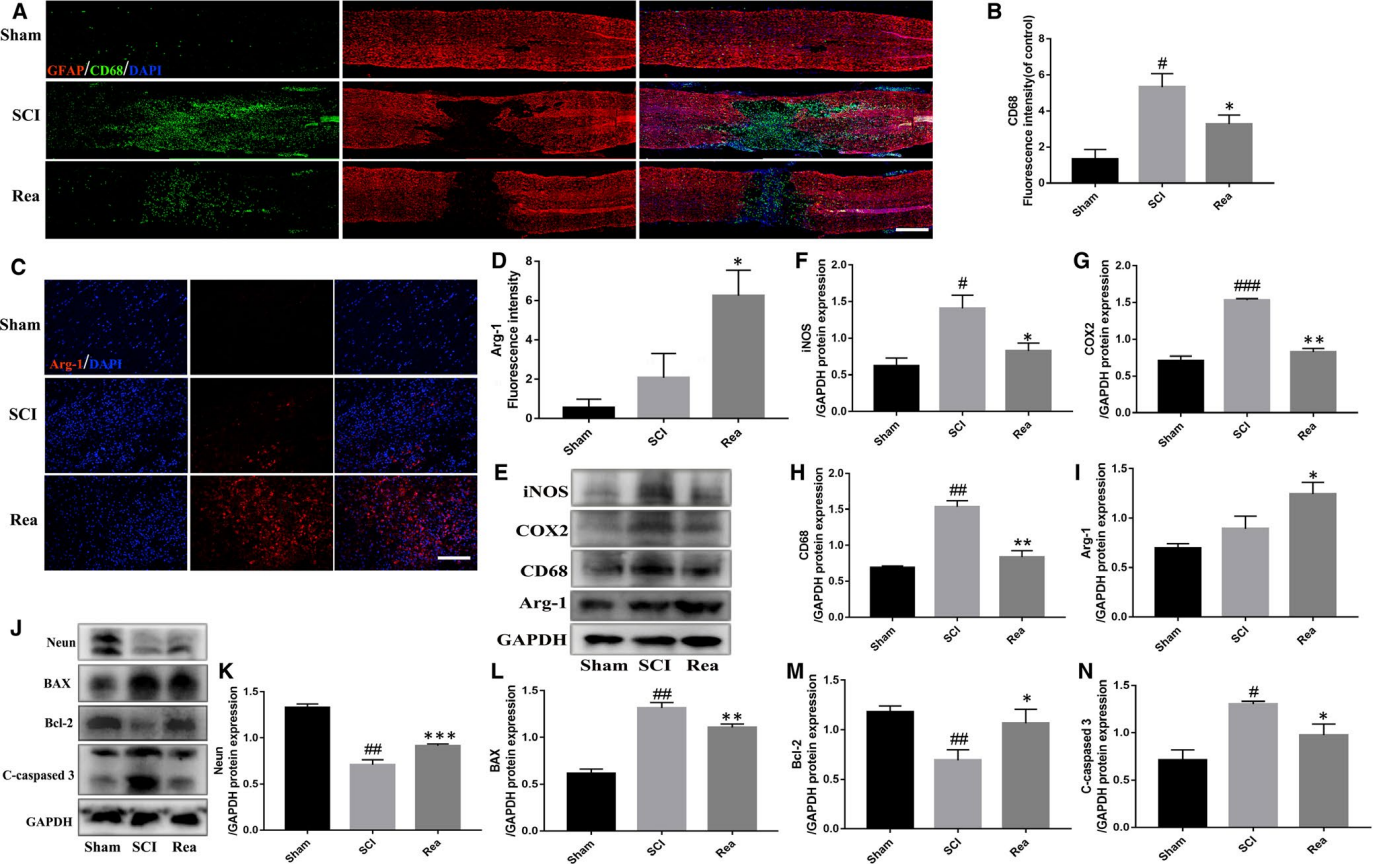
Rea inhibited the release of inflammation and attenuated neuron apoptosis in SCI rats. A total of 3 groups of rats were given Sham, SCI, SCI + Rea treatment. (A, B) Double‐fluorescence staining and quantification analysis for CD68 (green)/GFAP (red) of spinal cord tissue sections at 3 days post‐injury. Scale bar = 500 μm. (C, D) Representative images containing Arg‐1 immunofluorescence and quantification analysis on spinal cord sections at 28 days after SCI. Scale bar = 100 μm. (E‐I) Western blot results and quantification data of iNOS, COX‐2, CD68 and Arg‐1 proteins expression in each group of rats. (J‐N) Western blot results and quantification data of Neun, cleaved caspase‐3, BAX and Bcl‐2 proteins expression in each group of rats. Data are presented as the mean ± SD from three independent experiments. #*P* < .05, ##*P* < .01 vs the Sham group. *P < .05, **P < .01 vs the SCI group

**FIGURE 7:**
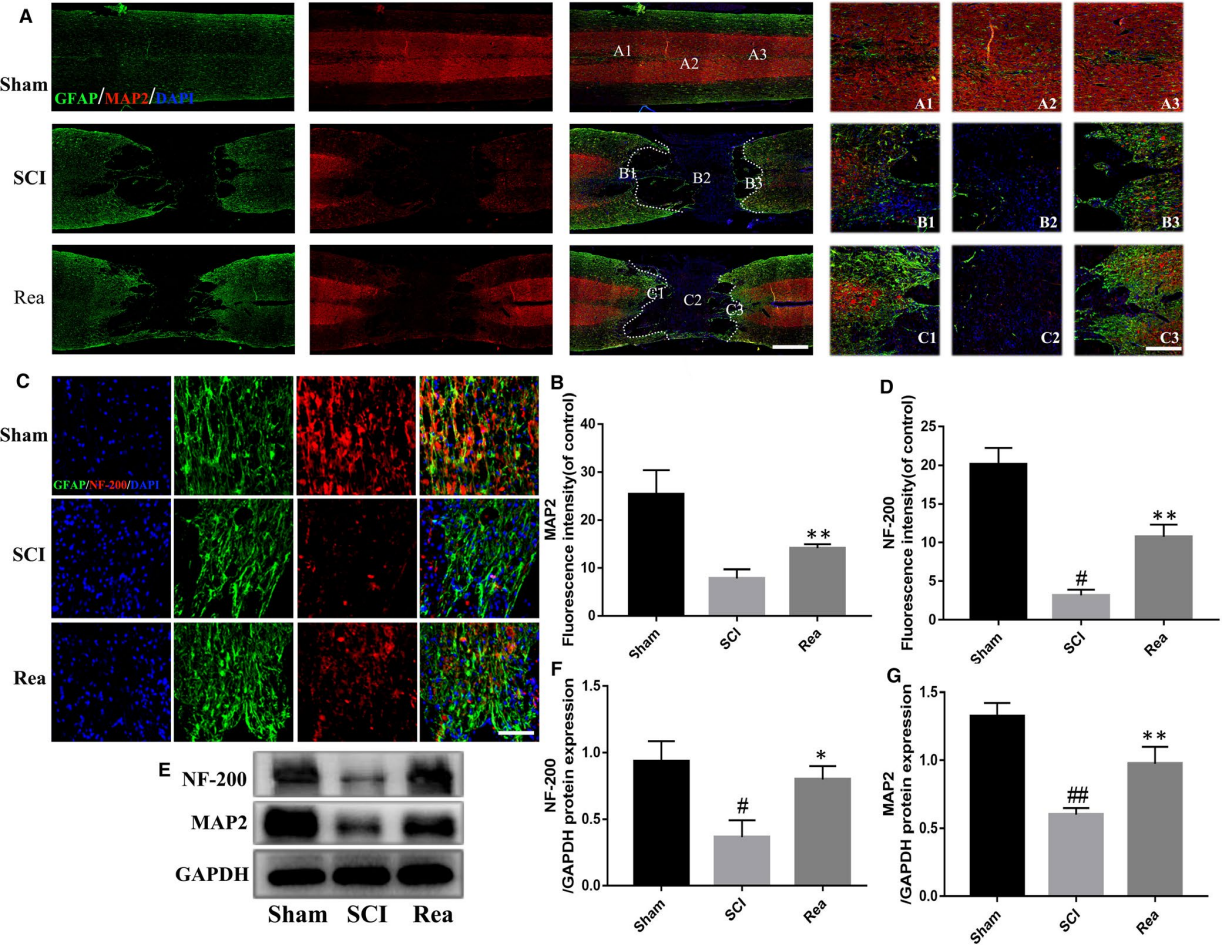
Rea enhances neural repair and axonal regeneration after SCI. (A, B) Double immunofluorescence images and quantification analysis showed MAP2 (red) and GFAP (green) at 28 d after SCI in each group. The dotted line indicates border of lesion site. Enlarged view of Sham, SCI and Rea group in the rostral area (A1, B1, C1), injury site (A2, B2, C2) and caudal area (C3, B3, C3) of spinal cords. Scale bar = 500 μm, amplification = 100 μm. (C, D) Representative images containing astrocytes and neurofilament (NF‐200) immunofluorescence and quantification analysis on spinal cord sections at 28 d after SCI. Scale bar = 100 μm. (E‐G) Western Blot protein expressions and quantification data of MAP2 and NF‐200 in each group. Data are presented as the mean ± SD from three independent experiments. #*P* < .05, ##*P* < .01 vs the Sham group, **P < .01 vs the SCI group

SCI triggers microglia/macrophage activation and polarization to the M1 phenotype, resulting in inflammatory damage that impairs tissue repair and aggravates neurological symptoms.^31^ Thus, re‐polarizing the activated microglia/macrophage to the anti‐inflammatory M2 phenotype can significantly improve recovery after SCI.^9^ The number and distribution of the M1 macrophages was determined by immunostaining with CD68 and GFAP. As shown in Figure 6A, both the density and spatial distribution of the CD68 + M1 microglia (green) in the injured spinal cord regions decreased by 41.4% following 28 days of Rea treatment. We also performed immunofluorescence for Arg‐1, a M2 microglia/macrophage marker. Interestingly, the numbers of M2 microglia/macrophage were increased after Rea treatment compared with SCI group (Figure 7C,D). Consistent with this and the in vitro findings, the spinal cord tissues of the Rea‐treated animals expressed significantly lower levels of CD68, iNOS and COX‐2 (Figure 6E‐H), suggesting that Rea can reduce the inflammatory response after SCI. Furthermore, the expression of Arg‐1 was also markedly increased after Rea treatment (Figure 6E,I), indicating that it can promote M2 polarization and suppress M1 polarization of the activated microglia/macrophage, which attenuates the inflammatory environment after SCI.

Several studies have reported a functional relationship between inflammation and neuronal apoptosis.^32^, ^33^ Sustained activation of microglia during pathological conditions also leads to neuronal apoptosis and necrosis, eventually impairing neurological functions.^11^ In addition, SCI also increased the expression of BAX and caspase‐3 and decreased that of Bcl‐2 compared to the Sham‐operated animals, all of which were restored to near normal levels by Rea treatment (Figure 6J,L,M,N). Rea also up‐regulated NeuN expression post‐SCI, which is indicative of neuronal survival (Figure 6J,K). Taken together, Rea can alleviate the inflammatory microenvironment in the spinal cord after physical trauma by promoting M2 polarization of the microglia, thereby reducing neuronal apoptosis and improving functional recovery after SCI.

### 3.6 Rea promoted neural repair and axonal mobilization after SCI

The effects of Rea on axon regeneration and axon repair were determined by immunostaining for MAP2, a structural protein of axonal microtubules. As shown in Figure 7A, the MAP2‐positive axons were sparsely distributed and showed low‐intensity staining in the SCI group. However, Rea treatment increased the density of the MAP2‐positive axons, which distinctly grew towards the injured area (Figure 7A). This suggested that Rea‐dependent recovery of injured axons and axon regeneration is the basis of the functional recovery after SCI. Furthermore, we observed few NF‐200 positive axons in the SCI group, whereas Rea‐treated animals showed increased number of NF‐200‐expressing nerve filaments (Figure 7C). Likewise, Rea treatment significantly increased the overall expression of MAP2 and NF‐200 (Figure 7E‐G) compared to the SCI group. Taken together, Rea can promote axon repair and regeneration, which is the cellular basis of the functional recovery after SCI.

## 4 DISCUSSION

**FIGURE 8:**
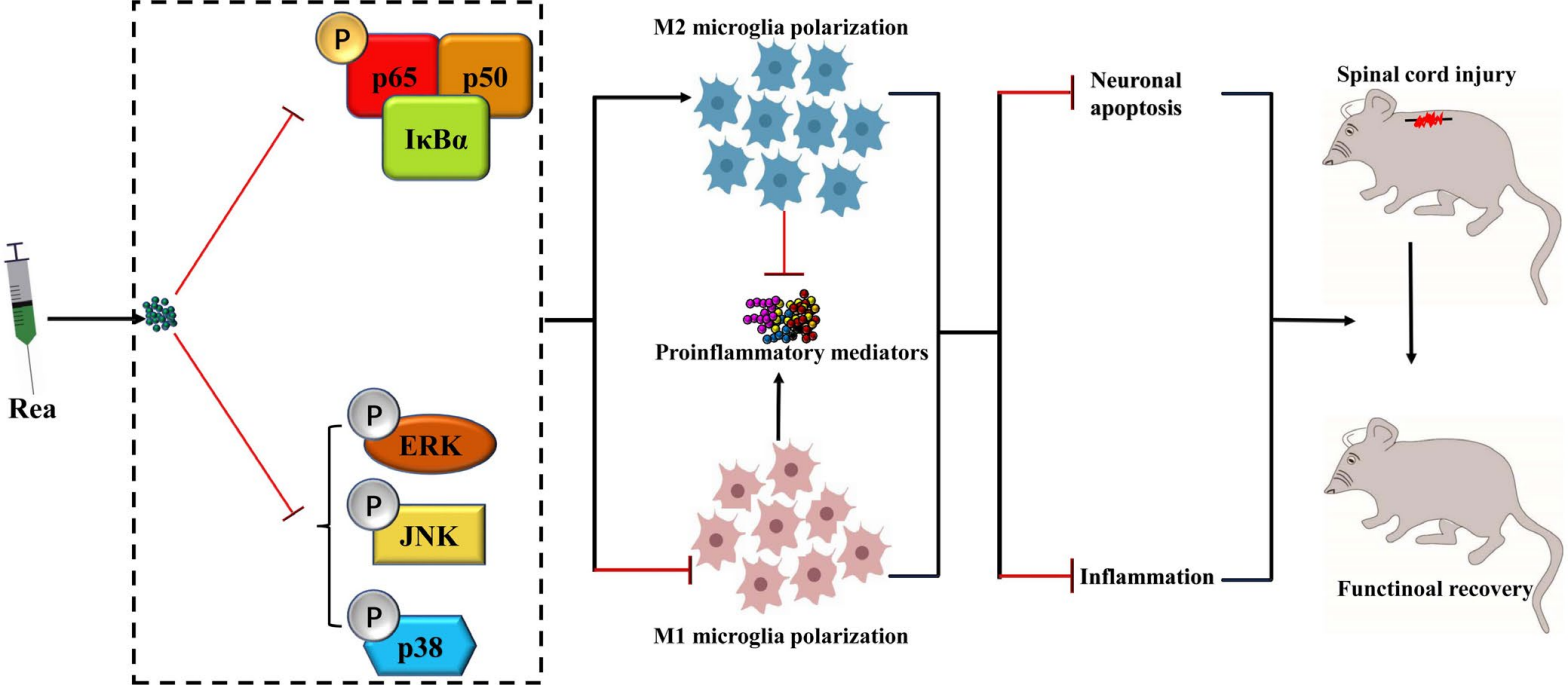
Schematic diagram of the effects of Rea on SCI. Rea treatment inhibits NF‐κB and MAPK signalling pathways to regulate microglia polarization and neuronal apoptosis, which promotes functional recovery of SCI rats

The incidence of spinal cord injury (SCI) due to accidents and other physical trauma has increased in recent years, and warrants novel, effective therapeutic strategies.^34^, ^35^, ^36^ Several Chinese herbal medicine formulations have shown significant therapeutic effects on SCI.^37^, ^38^ Rea is a neuroprotective and anti‐inflammatory compound present in the herbal extract of *R radix*, which is used to treat neurodegenerative diseases. However, no study so far has tested the potential therapeutic effects of this extract against SCI. We found that Rea inhibited LPS‐induced release of inflammatory factors from the microglial cell line BV2 and polarized the cells to the M2 phenotype while suppressing the M1 phenotype. In addition, the anti‐inflammatory action of Rea inhibited apoptosis in the neurons co‐cultured with the BV2 cells by blocking the NF‐κB and MAPK pathways. Finally, Rea ameliorated the secondary injury after SCI in a rat model and promoted locomotor function recovery. The secondary injury after SCI^39^ involves extensive neuroinflammation and neuronal apoptosis,^40^, ^41^ and is the primary target of SCI therapy. However, most therapies mitigate either of the two pathological processes. According to our results, we demonstrate that Rea on the other hand not only suppressed the release of inflammatory mediators from LPS‐stimulated microglia by targeting the NF‐κB and MAPK pathways, but also inhibited apoptosis in the co‐cultured neuronal cells (Figure 8). Therefore, it can reduce the secondary injury after SCI and promote the recovery of its motor function.

Microglia are the innate immune cells of the central nervous system, and the hyperactivated microglial cells promote inflammatory responses during pathological conditions, leading to neuronal apoptosis and tissue damage.^42^, ^43^, ^44^ We established an in vitro model of neuroinflammation by co‐culturing the neuronal PC12 cell line with the LPS‐stimulated microglial BV2 cell line.^38^, ^45^ The LPS‐activated BV2 cells produced significantly higher levels of iNOS and COX‐2 compared to the untreated controls, which in turn up‐regulated the downstream targets including NO and PGE2,^18^ thereby enhancing the response associated with acute and chronic inflammation. Rea significantly decreased the expression levels of these inflammatory mediators, indicating that it can mitigate neuroinflammation during SCI and prevent the ensuing tissue damage.

Polarization of microglia plays the vital role in the initiation of neuroinflammation after SCI. Microglial cells can be fully activated with the M1 phenotype or M2 phenotype,^46^ and the switching between M1 and M2 affects the final inflammatory response. After SCI, most microglial cells are polarized to the M1 phenotype, leading to the release of pro‐inflammatory mediators such as iNOS, COX‐2, TNF‐α and IL‐1β. These factors prolong and aggravate the neuroinflammation process, resulting in the death or dysfunction of neighbouring neurons, which is not conducive to SCI repair.^47^, ^48^ Therefore, a viable therapeutic strategy for SCI is to dampen the inflammatory response by promoting microglial polarization to the M2 phenotype. The anti‐inflammatory M2 microglial cells release CD206, arginase (ARG) ‐1, IL‐4, IL‐10 and neurotrophic proteins which increase neuronal survival and promote tissue regeneration. In our study, we demonstrated that Rea inhibited M1 polarization of microglia in vitro and promoted M2 polarization (Figure 2). We also evaluated the effect of Rea pre‐treatment on the apoptosis of PC12 cells co‐cultured with LPS‐stimulated BV2 cells. Rea pre‐treatment restored the viability of PC12 cells, indicating that it can enhance the survival of neurons and decrease apoptosis. SCI induction in the rat model significantly increased the density of the CD68 + M1 microglia/macrophage. Rea treatment markedly lessened the number of M1 microglia/macrophage, especially in the damaged lesion, and prevented their spread. Furthermore, the expression of CD68, iNOS and COX‐2 in the damaged region also decreased following Rea treatment in vivo. In addition, the frequency of the NeuN + neurons also recovered after Rea treatment compared to the untreated SCI group. Taken together, Rea can significantly reduce neuronal apoptosis by inhibiting neuroinflammation and alleviate the damage caused by SCI.

The NF‐κB and MAPK pathways are the key regulators of inflammation‐related genes.^49^ We hypothesized therefore that Rea exerts is anti‐inflammatory effects by targeting the NF‐κB and MAPK signalling pathways. NF‐κB is a transcription factor that is activated by endotoxins, bacteria, cytokines, tumour antigens etc,^20^, ^50^ which then translocates to the nucleus and promotes expression of pro‐inflammatory genes like iNOS, COX‐2, IL‐6, IL ‐1β etc Rea inhibited NF‐κB activity by simultaneously inhibiting p65 transcription and phosphorylation even at the low concentration of 20µM. MAPKs also lie upstream of the inflammatory cascade^24^, ^51^ and activate the ERK, JNKs and p38 pathways.^19^, ^52^, ^53^ Subsequently, these transcription factors activate downstream genes to promote the release of pro‐inflammatory factors, leading to neuroinflammation. Rea treatment significantly decreased the phosphorylation levels of NF‐κB p65, I‐κBα, JNK, ERK and p38 in a dose‐dependent manner. Therefore, its neuroprotective effects can be attributed to the inhibition of LPS‐induced NF‐κB and MAPK signalling.

To summarize, Rea can mitigate neuroinflammation and promote M2 polarization of microglia by inhibiting NF‐κB and MAPK signalling pathways, thereby reducing neuronal apoptosis and improving functional recovery after SCI. Therefore, the NF‐κB and MAPK cascades are suitable targets for reducing inflammation during neurological injuries. However, the long‐term side effects of Rea remain uncertain and there is a need to develop novel delivery systems for sustained and targeted drug release in order to improve the therapeutic outcomes.^54^, ^55^ The mechanistic basis of Rea action also needs to be explored further.

## 5 CONCLUSION

Rea inhibited LPS‐induced inflammation by targeting the NF‐κB and MAPK signalling pathways, and prevented neuronal apoptosis by promoting M2 polarization of microglia/macrophage in vivo and in vitro. The above research prove that Rea may become a potential drug for SCI therapy.

## CONFLICT OF INTEREST

The authors confirm that there are no conflicts of interest.

## AUTHOR CONTRIBUTION

**Shining Xiao:** Data curation (equal). **Chenggui Wang:** Formal analysis (equal). **Quanming Yang:** Investigation (equal). **Haibin Xu:** Methodology (equal). **Jinwei Lu:** Investigation (equal). **Kan Xu:** Data curation (lead); Funding acquisition (lead).
